# Exploring and analyzing the role of hybrid spectrum sensing methods in 6G-based smart health care applications

**DOI:** 10.12688/f1000research.144624.2

**Published:** 2024-05-23

**Authors:** Arun Kumar, Raminder Kaur, Nishant Gaur, Aziz Nanthaamornphong

**Affiliations:** 1 Department of Electronics and Communication Engineering, New Horizon College of Engineering, Bangalore, India; 2 Department of CSE, JECRC University, Jaipur, India; 3 Department of Physics, JECRC University, JECRC U, India; 4 College of Computing, Prince of Songkla University, Phuket Campus, Thailand

**Keywords:** Smart hospitals, Spectrum Sensing, Cognitive Radio, Hybrid Spectrum sensing

## Abstract

**Background:**

Researchers are focusing their emphasis on quick and real-time healthcare and monitoring systems because of the contemporary modern world’s rapid technological improvements. One of the best options is smart healthcare, which uses a variety of on-body and off-body sensors and gadgets to monitor patients’ health and exchange data with hospitals and healthcare professionals in real time. Utilizing the primary user (PU) spectrum, cognitive radio (CR) can be highly useful for efficient and intelligent healthcare systems to send and receive patient health data.

**Methods:**

In this work, we propose a method that combines energy detection (ED) and cyclostationary (CS) spectrum sensing (SS) algorithms. This method was used to test spectrum sensing in CR-based smart healthcare systems. The proposed ED-CS in cognitive radio systems improves the precision of the spectrum sensing. Owing to its straightforward implementation, ED is initially used to identify the idle spectrum. If the ED cannot find the idle spectrum, the signals are found using CS-SS, which uses the cyclic statistical properties of the signals to separate the main users from the interference.

**Results:**

In the simulation analysis, the probability of detection (Pd), probability of a false alarm (Pfa), power spectral density (PSD), and bit error rate (BER) of the proposed ED-CS is compared to those of the traditional Matched Filter (MF), ED, and CS.

**Conclusions:**

The results indicate that the suggested strategy improves the performance of the framework, making it more appropriate for smart healthcare applications.

## 1. Introduction

Sixth-generation (6G)-based smart hospitals require substantial spectrum resources for ultra-reliable and low-latency communications. Utilizing a diverse range of frequency bands, including millimeter waves (mm) and terahertz frequencies, is crucial to support real-time medical data exchange, remote surgeries, and AI-driven healthcare applications, ensuring the highest level of patient care and safety. ^1^^,^^2^ The spectrum is vital for wireless applications because it provides essential electromagnetic frequencies for transmitting data wirelessly. It determines the data capacity, speed, and coverage. Efficient spectrum allocation ensures reliable communication, supports the growing demand for wireless services, and enables innovations such as the fifth generation (5G), the internet of things (IoT), and wireless broadband to enhance connectivity and productivity. Obtaining high spectral access faces challenges owing to spectrum scarcity and allocation inefficiencies. The crowded frequency bands, legacy systems, and regulatory constraints limit the available spectrum. Efficient sharing mechanisms, interference management, and international coordination are needed to optimize spectral access, enabling the growth of wireless technologies, such as 5G and beyond. Healthcare applications encounter significant challenges regarding spectrum availability and efficiency. The increasing demand for wireless healthcare services exacerbates spectrum congestion, leading to potential interference and degraded performance. Moreover, strict regulatory constraints limit the available spectrum for medical use. Hybrid spectrum sensing presents a promising solution by combining multiple sensing techniques, such as energy detection, matched filtering, and cyclostationary feature detection. By leveraging the strengths of each method, hybrid sensing enhances spectrum awareness, enabling healthcare systems to detect and utilize idle spectrum more effectively. This approach also improves spectrum utilization efficiency by accurately identifying and dynamically accessing available spectrum resources while mitigating interference risks. Additionally, hybrid spectrum sensing enhances reliability by providing robust detection in diverse environmental conditions and mitigating the effects of fading and shadowing. Overall, hybrid spectrum sensing offers a comprehensive solution to address the spectrum-related challenges faced by healthcare applications, ensuring reliable and efficient wireless connectivity for critical medical services. ^3^ Spectrum sensing (SS) makes 5G-based healthcare apps more dependable and effective by facilitating more effective data transmissions. By enabling devices to recognize and make use of available frequency bands, interference is reduced, and dependable connectivity is guaranteed. This is necessary for telemedicine, remote surgery, and real-time monitoring where continuous data flow is essential. By providing priority access to medical records, spectrum sensing helps in emergency scenarios by guaranteeing patient safety. In conclusion, it improves network efficiency, lowers latency, and increases the standard and accessibility of healthcare services in the context of 5G. Cognitive radio-based spectrum sensing plays a pivotal role in achieving high spectral access by enabling dynamic and intelligent spectrum allocation. These systems continuously monitor the radio frequency environment and detect unused or lightly utilized spectral bands. When such spectrum opportunities are identified, cognitive radios can instantly adapt their transmissions to access these frequencies and optimize their spectrum utilization while minimizing interference. This technology enhances spectral efficiency, accommodates more users, and fosters better coexistence among wireless services, ultimately increasing overall spectral access and network performance. ^4^ Energy detection (ED) is a spectrum-sensing technique that determines the presence of a signal by measuring the power within a given frequency band. It is a simple and widely used method suitable for detecting signals with unknown characteristics. Matched filter (MF) is a signal-processing filter optimized to maximize the signal-to-noise ratio (SNR) when a known signal is present in noise. It is particularly effective when the signal shape is known, making it useful in scenarios where signal patterns are well defined, such as radar or communication systems. Cyclostationary (CS) spectrum sensing analyzes statistical periodicities in the received signal, exploiting CS features, such as symbol rate, to detect and identify signals. This method is advantageous when dealing with modulated signals because it provides improved signal detection and interference mitigation in dynamic spectrum environments. Conventional SS algorithms, such as ED, MF, and CS, are fundamental techniques used in cognitive radio systems to detect and utilize the available spectrum opportunistically while avoiding interference with primary users. These algorithms serve as the foundation for dynamic spectrum access. These conventional spectrum sensing algorithms are valuable for enabling cognitive radiologists to make informed decisions about spectrum usage. In the framework of 6G networks, spectrum sensing is essential for developing smart healthcare applications. 6G-enabled healthcare systems may dynamically distribute spectrum resources to medical equipment, guaranteeing uninterrupted connectivity and real-time data transfer, by effectively identifying and analyzing underutilized or unused spectrum bands. This feature makes it easier for telemedicine, IoT devices for healthcare, and remote patient monitoring to proliferate, promoting prompt and accurate healthcare delivery. Additionally, spectrum sensing improves spectrum reliability and efficiency, which is essential for enabling the ultra-low latency and large data rates needed by developing healthcare applications in 6G networks. ^5^ However, each has limitations that researchers continue to address through advancements in cognitive radio technology, signal processing techniques, and machine learning to improve spectrum-sensing accuracy, adaptability, and efficiency in dynamic and complex wireless environments. ^6^ The suggested article is organized as follows: the article’s background, motivation, and contributions are covered in Section 1, related work is covered in Section 2, a system model is shown in Section 3, and the simulated outcomes and the proposed article’s conclusion are covered in Sections 4 and 5.

### 1.1 Motivation

SS plays a crucial role in enhancing healthcare applications across various scenarios by ensuring reliable and efficient wireless communication. Here are different scenarios where spectrum sensing benefits healthcare ^7^^–^^9^:

• Emergency Response and Disaster Recovery: During natural disasters or emergencies, dedicated spectrum sensing helps prioritize and allocate spectrum resources for healthcare providers. This ensures that first responders and medical teams have uninterrupted communication for coordinating rescue efforts and providing critical medical assistance, even in congested or disrupted networks.
• Wireless Medical Devices: Spectrum sensing is essential for wireless medical devices like remote patient monitors, wearable health trackers, and smart healthcare implants. These devices rely on spectrum sensing to identify the most suitable and interference-free frequency bands, ensuring real-time data transmission and accurate patient monitoring.
• Telemedicine: Spectrum sensing is vital for telemedicine applications, where doctors and patients communicate remotely. It enables healthcare professionals to maintain consistent and high-quality video and audio connections, even in crowded wireless environments. This ensures that telemedicine consultations and remote diagnostics are conducted without interruptions, improving the accessibility of healthcare services.
• IoT Healthcare Devices: The IoT has revolutionized healthcare with devices such as smart pill dispensers, medication trackers, and patient location monitors. Spectrum sensing ensures these IoT devices can operate in less congested bands, facilitating the reliable transmission of health-related data to healthcare providers and caregivers.
• Wireless Imaging and Diagnostics: Healthcare facilities increasingly rely on wireless technologies for transmitting large medical imaging files, including MRIs, CT scans, and X-rays. Spectrum sensing helps allocate suitable spectrum resources, ensuring that these critical files are transmitted efficiently and promptly, allowing for quicker diagnosis and treatment planning.
• Mobile Clinics: Spectrum sensing is beneficial for mobile healthcare clinics that operate in remote or underserved areas. These clinics can use spectrum sensing to identify the best available spectrum for their wireless communication needs, ensuring that healthcare professionals can access patient records, medical databases, and telemedicine consultations without network disruptions.
• Healthcare Robotics: Spectrum sensing is essential for healthcare robotics, including robotic surgical systems and remote-controlled medical robots. It ensures precise and real-time control during medical procedures, where any signal interference or delay can have critical consequences.
• Ambulance Communication: Ambulances equipped with advanced medical equipment and communication systems rely on spectrum sensing. It helps these vehicles maintain connectivity, allowing paramedics and emergency medical personnel to transmit patient information, vital signs, and other critical data to hospitals, ensuring seamless patient care transitions.

### 1.2 Contributions

Spectrum scarcity is a problem that cognitive radio (CR) is designed to address for various wireless services and applications. Smart healthcare solutions that use CR technology can transmit and receive monitoring data over the primary user (PU) frequency range without interfering with PU transmission. Smart healthcare based on CR solves two fundamental problems. First, based on the findings of spectrum sensing, it enables monitoring sensors (secondary users) to wirelessly communicate data while the PU does not use the spectrum. Second, it increases the effectiveness of spectrum usage. ^10^ Implementing a proposed hybrid approach such as the ED-CS and DMF techniques for 6G-based smart hospital applications offers several valuable contributions to the healthcare industry.

• The hybrid approach provides superior SS by leveraging the sensitivity of ED to detect energy in a frequency band and the precision CS to confirm the presence of specific signals. This results in a comprehensive and accurate view of the spectrum environment within the smart hospital. High probability of detection in 6G-based smart healthcare applications is crucial for seamless, ultra-reliable connectivity. These algorithms minimize interference, enabling real-time, high-quality data transmission from medical devices. This enhances telemedicine, remote patient monitoring, and precision diagnostics, ultimately improving healthcare accessibility and patient outcomes in the advanced 6G era.
• The hybrid ED-CS ensures the reliable detection of wireless medical devices and sensors. This is crucial for monitoring patients’ vital signs, enabling timely interventions, and enhancing patient safety. With accurate spectrum sensing, smart hospitals can optimize their wireless networks and devices, leading to improved operational efficiency, reduced downtime, and cost savings.
• As 6G technology evolves, the hybrid approach ensures that smart hospitals are well-equipped to adapt to the increasing demands of advanced wireless communication. It future-proofs healthcare infrastructure, allowing hospitals to stay at the forefront of technology by enhancing the throughput of the framework. High-throughput SS algorithms in 6G-based smart healthcare applications facilitate rapid data transmission, supporting real-time remote monitoring and medical data exchange. This enables complex diagnostic procedures, telemedicine, and seamless healthcare services, offering enhanced patient care and health outcomes in the high-speed, low-latency 6G environment.
• Real-time and accurate spectrum sensing enhances patient care by enabling continuous monitoring and data transmission from medical devices. This contributes to early detection of medical issues, faster response times, and better overall patient outcomes.

## 2. Literature review

The integration of hybrid spectrum sensing methods into 6G-based smart healthcare systems offers a multitude of benefits. Firstly, it enhances spectrum efficiency by intelligently utilizing available spectrum resources, ensuring optimal allocation for healthcare applications amidst growing demand and spectrum scarcity. Secondly, hybrid sensing improves reliability by mitigating interference and dynamically adapting to changing wireless environments, thereby enhancing the quality and consistency of healthcare data transmission. Thirdly, it enables seamless connectivity and coverage extension, crucial for supporting remote patient monitoring, telemedicine, and IoT devices in diverse healthcare settings. Additionally, hybrid spectrum sensing enhances security and privacy by identifying and mitigating potential threats and unauthorized access attempts. Moreover, by optimizing spectrum usage and improving connectivity, it facilitates innovative healthcare services and applications, fostering advancements in medical diagnostics, treatments, and patient care delivery. Overall, the integration of hybrid spectrum sensing into 6G-based smart healthcare systems promises to revolutionize healthcare delivery, ensuring efficient, reliable, and secure wireless communication for improved patient outcomes. The literature review on SS in this section can give a thorough understanding of the research, methods, and developments in this area. Table 1 presents a validation of the proposed technique by comparing it with previous studies in the literature. This comparison serves as a benchmark to evaluate the effectiveness and performance of the proposed approach in addressing similar problems:

**Table 1.** Summary of spectrum sensing methods and research findings in the literature review.

References	Remark
^11^	In this paper, a hybrid detector that combines cyclostationary (CS) and energy detection (ED) is used to determine the status of the primary user (PU) in a cognitive radio network (CRN). Preferably, ED is primarily used for spectrum sensing because of noise uncertainty and its simple computational architecture. However, the intricacy of the framework has not been discussed and analyzed.
^12^	A hybrid detector (HD) is suggested in this research to identify the spectrum hole using the available spectrum resources. An ED and a MD serve as the foundation for HD design. HD can sense the signal more precisely than a single detector like an ED or MD. Whether information about the PU is available or not, HD can operate in both scenarios. In the CSS environment, the probability of detection (Pd) is increased to 1 and the probability of a false alarm (Pfa) is decreased to 0 under low SNR conditions at 20 dB SNR. The results also indicate that the proposed algorithm may improve the hardware complexity of the framework. The proposed Hybrid spectrum sensing can be complex, requiring significant computational resources and may not perform well in dynamic environments.
^3^	This study presents a new 5G HMF algorithm based on the integration of two MFs that was used to construct a spectrum sensing technique. We also contrasted the HMF's performance in Rayleigh and Rician channels, as well as conventional MF. The HMF has been seen to work more efficiently on both channels than the traditional MF. The use of two MF spectrum sensing is susceptible to higher computational complexity, as it involves processing the signal twice, which can be resource-intensive in real-time applications and may lead to increased power consumption.
^13^	Using multi-core processors, the authors of this work suggested an architecture for spectrum sensing that permits a decrease in the computational time required to obtain the CS properties of a signal. The proposed architecture may achieve over 92.8% parallel efficiency, according to simulation data, which reduces the time required for spectrum sensing by a factor of 29.7. Utilizing multi-core processors for spectrum sensing can lead to increased power consumption and heat generation, potentially limiting the battery life of portable devices and requiring advanced cooling solutions in resource-constrained environments.
^14^	The effectiveness of such a hybrid SS is investigated, as is the effect of the novelty detection model parameters on the detection performance. These simulations do, in fact, support the effectiveness of the hybrid SS system's proposed one-class learning method. The proposed methods can be complex to implement and may require a combination of hardware and software, making them less cost-effective and challenging to standardize in diverse communication systems.
^15^	The authors proposed a novel CS method for 5G waveforms. The complexity of CS is decreased by limiting the computation of CS features and the signal autocorrelation. The ED and CS spectrum sensing methods based on CR are described to assess the performance of 5G waveforms. The study's findings demonstrate that the suggested CS algorithm performed well in terms of detection and gained 2 dB in comparison to the industry norms.
^16^	The suggested algorithm's performance was evaluated in the low SNR regime with noise uncertainty over a variety of fading and non-fading channels. The simulation studies' findings showed that spectrum sensing performance had improved in accordance with the best threshold selection. However, the estimation of dynamic threshold in various channel condition may upsurge the complexity of the framework.
^17^	For cognitive radio networks with numerous terminals and a single fusion center, the authors presented a cooperative spectrum sensing technique. It has been demonstrated that the suggested technique performs consistently even at low SNR levels. Then, simulation findings are offered to support the suggested studies. The presented approach can introduce overhead due to information exchange among nodes, may be vulnerable to security breaches, and can suffer from synchronization issues among distributed sensors, impacting system reliability.
^18^	The method for hybrid spectrum sensing in CR systems using soft computing paradigms is described in this study as being updated and effective. The proposed soft computing approach uses an artificial neural network for learning and decision-making as a solution to the issues that arise when a new product is put through the CR framework. It also developed mechanisms that allow unlicensed cognitive users to access radio frequencies through a spectrum hole and comprehend their implications.
^19^	In the proposed study, the authors concentrated on implementing cutting-edge waveforms such as the filter bank multi-carrier (FBMC) system, universal filter multi-carrier (UFMC), and non-orthogonal multiple access (NOMA). The work analyzed and studied a number of factors, including PSD, BER, capacity, and PAPR of advanced waveforms and OFDM techniques.
^20^	In the proposed work, secondary users and nodes (SUs) in a single cluster are dynamically correlated with each other according to their statistical behavior when engaging in smart cooperative communication in CRSNs to increase the viability of Internet of Things (IoT) applications for the connected world. The use of distributed artificial intelligence (DAI). Based on their individual coordinator agents (CoA), determine the real-time resource distribution to these clusters. On the dynamism of the behaviors. To increase sustainability in smart city applications, the pre-A reduction in the number of unused channels makes these applications more energy efficient.
Proposed Work	To the best of our knowledge and related works, so far, the exploration of SS methods in smart healthcare applications has not been implemented or presented. In this work, we focused on the utilization of hybrid SS algorithms, namely ED and CS, for enhancing the performance of several parameters such as BER, Pfa, Pd, and PSD. Further, the proposed work also explores the use of SS methods to improve the smart healthcare service provided by the 5G/6G radio system.
^21^	In the proposed work, SUs in a single cluster is dynamically correlated with each other according to their statistical behavior when engaging in smart cooperative communication in CRSNs to increase the viability of IoT applications for the connected world. The usage of Distributed Artificial Intelligence based on their individual Coordinator Agents, determine the real-time resource distribution to these clusters. On the dynamism of the behaviors. To increase sustainability in smart city applications, the pre-Reduction in the number of unused channels makes these applications more energy efficient.

## 3. Methods

**Figure 1.**
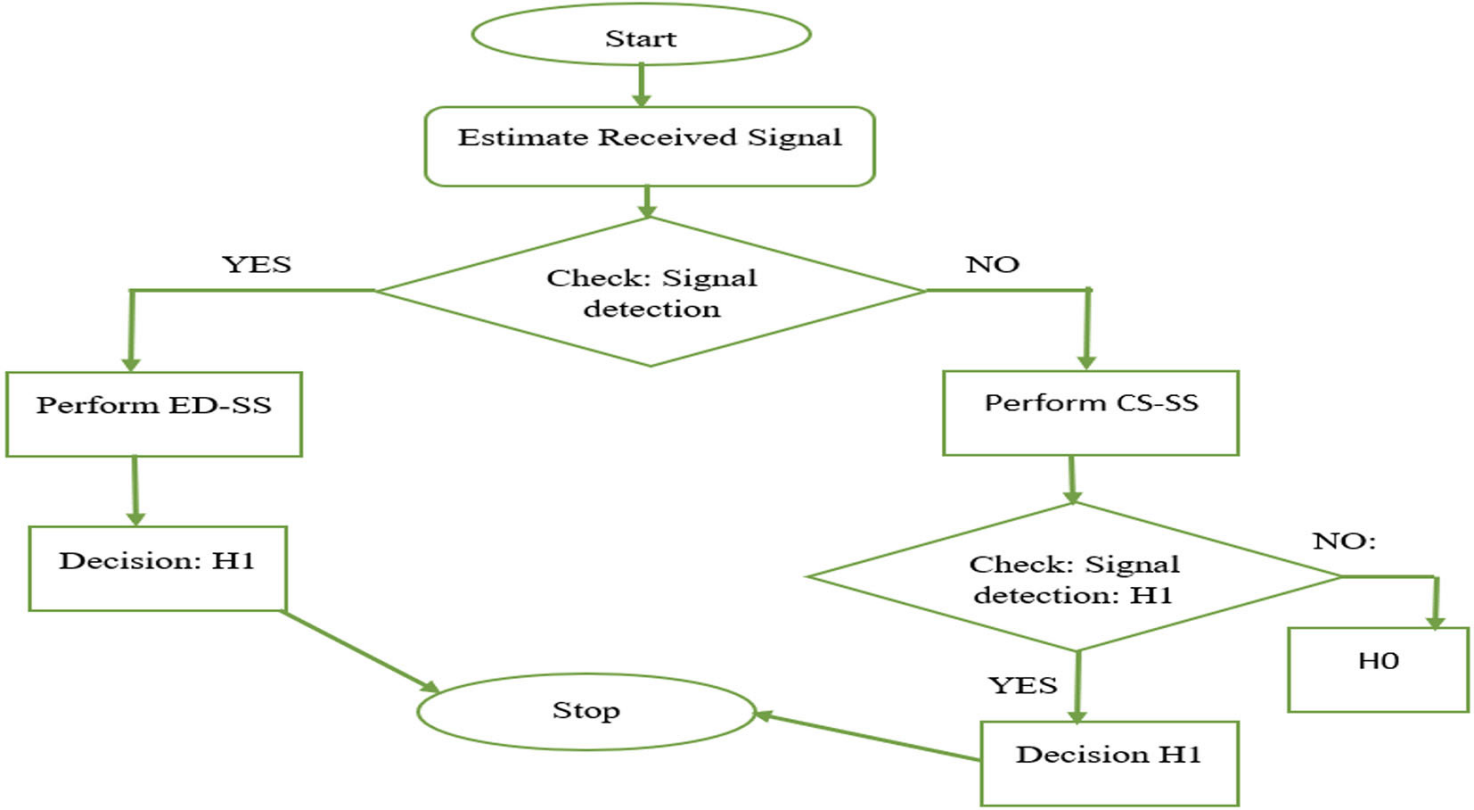
Proposed energy detection-cyclostationary spectrum (ED-CS).

The proposed hybrid SS combines ED and CS techniques for spectrum sensing in cognitive radio systems. ED measures the power of the received signal, while CS detection analyzes the cyclic statistical properties. Hybrid spectrum sensing, which combines ED and CS analysis, can significantly enhance healthcare services. ED ensures the detection of wideband signals, whereas CS analysis identifies specific signal characteristics, enhances signal reliability, and reduces false alarms. In healthcare, this approach can improve the quality of service by ensuring the reliable and timely transmission of critical medical data, optimizing resource allocation, and reducing interference. For telemedicine, remote monitoring, and emergency response, the hybrid technique offers a robust and efficient solution that provides consistent connectivity and minimizes the risk of data loss or delays, ultimately leading to improved patient care and safety in 5G-based healthcare applications. In this approach, the received signal is first processed using ED to quickly detect the presence of the primary signal. If energy-based detection is inconclusive, CS detection is applied to exploit signal-specific cyclic features. This hybrid approach enhances detection accuracy, especially in low signal-to-noise ratio (SNR) scenarios, making it suitable for dynamic spectrum access, where reliable primary signal detection is critical for efficient spectrum sharing. A diagram of the proposed Ed-CS is shown in Figure 1.

The detection performance of ED is analyzed in the following conditions:

$$h_{0}=z\left(n\right)=\sigma_{n}$$ (1)

$$h_{1}=z\left(n\right)=x\left(n\right)+\sigma_{n}$$ (2)

Equations (1) and (2) indicate the non-availability and availability of the Primary User (PU), *z*( *n*) is the original signal, *x*( *n*) is the transmitting signal, and $\sigma_{n}$ is the noise variance in the Rician channel. The threshold estimation is given by

$$T_{\mathrm{Eth}}=\sum_{m=0}^{M-1}{\left|z\left(m\right)\right|}^{2}$$ (3)

where, *m* denotes the number of samples. Furthermore, Equations (1) and (2) can be formulated using the central limit theorem given by

$$h_{0}:T_{\mathrm{st}}\;\mathrm{Nor}\left(Mw_{m}^{2}+2Mw_{x}^{4}\right)$$ (4)

$$h_{1}=T_{\mathrm{st}}\;\mathrm{Nor}\left(\left(w_{m}^{2}+w_{x}^{2}\right),2M{\left(w_{m}^{2}+w_{x}^{2}\right)}^{2}\right)$$ (5)

where $T_{\mathrm{st}}$ is the distribution of the M statistic samples. The $T_{\mathrm{st}}$ was estimated and compared with the dynamic threshold value ( $\lambda_{D})$ to indicate the presence and absence of the idle spectrum. Furthermore, to estimate the performance of the ED, parameters such as probability of detection ( *P* *_d_*), probability of false alarm ( *P* *_fa_*), and power spectral density (PSD) should be analyzed.

$$P_{d}=\text{Prob}\left(T_{\mathrm{st}}>\frac{\lambda_{D}}{h_{1}}\right)=Q\left(\frac{\lambda_{D}-M\left(w_{m}^{2}+w_{x}^{2}\right)}{\sqrt{2M{\left(w_{m}^{2}+w_{x}^{2}\right)}^{2}}}\right)$$ (6)

$$P_{\mathrm{fa}}=\text{Prob}\left(T_{\mathrm{st}}>\frac{\lambda_{D}}{h_{0}}\right)=Q\left(\frac{\lambda_{D}-Mw_{m}^{2}}{\sqrt{2M}(w_{m}^{4})}\right)$$ (7)

The factor *Q*(.) is defined as:

Qs=12π∫S∞exp−0.5z2ds (8)

The PSD of the signal *z*( *n*) is given by:

$$S\left(f_{k}\right)=\frac{1}{N}{\left|\sum_{m=0}^{M-1}z\left(m\right)\exp\left(-j2\pi f_{k}m\right)\right|}^{2}$$ (9)

where $f_{k}$ is the frequency at which the PSD is computed, *N* is the signal length, and *S*( *f*_*k*_) is the PSD at frequency $f_{k}$ . Initially, the proposed ED detects the signal; otherwise, the CS algorithm is used. Let us consider the CS signal *z*( *t*). The mean and autocorrelation of *z*( *m*) are given by:

$$\text{Mean}\;z\left(t\right)=E\left\{z\left(t\right)\right\}$$ (10)

$$R_{z}\left(t,\tau \right)=\sum_{\beta }R_{z}^{\beta }\;\exp\left(j,2,\mathrm{\pi \beta t}\right)$$ (11)

The *P*_*d*_ and *P* *_fa_* for the CS is given by:

$$P_{d}=\text{Prob}\left(T_{\mathrm{st}}>\lambda_{D}\right)$$ (12)

$$P_{\mathrm{fa}}=\text{Prob}\left(\text{False Alarm}\right)$$ (13)

To calculate *P*_*fa*_, when only noise or noncyclostationary interference is present, the threshold ( $\lambda_{D})$ is utilized as follows:

$$P_{\mathrm{fa}}=\text{Prob}\left(T_{\mathrm{st}}>\lambda_{D}\right)$$ (14)

The PSD of the CS is related to the cyclic autocorrelation function by taking the Fourier transform with respect to the cyclic frequency ( β) , given as

$$S_{x}\left(f\right)=\int_{-\infty }^{\infty }R_{z}\left(\tau ,\beta \right)\exp\left(-j2\mathrm{\pi f\tau}\right)$$ (15)

To enhance the efficiency of CR, we propose a hybrid SS algorithm known as ED-CS. In the proposed algorithm, the ED estimates the availability of the PU. If the ED fails to estimate the occurrence of a PU, then the CS method is used to detect the PU. Hence, it is concluded that CS is used if the ED is unable to detect the PU. Therefore, the Pd for the cognitive user undergoing CS-SS is given by

$$P_{d-\mathrm{CS}}=1-P_{d-\mathrm{ED}}$$ (16)

$$P_{d-\mathrm{CS}}=P_{\mathrm{Md}-\mathrm{ED}}$$ (17)

The probability that cognitive user undertake CS is given by:

$$\text{prob}\left(k\right)=\sum_{k=0}^{M}\left(\begin{array}{c} M\\ K \end{array}\right)\;{\left(P_{d-\mathrm{CS}}\right)}^{K}{\left(1-P_{d-\mathrm{CS}}\right)}^{M-K}$$ (18)

Equation (15) denotes the prospect that the *K*-cognitive user out of *M* is subjected to the CS spectrum-sensing approach.

## 4. Results

**Figure 2.**
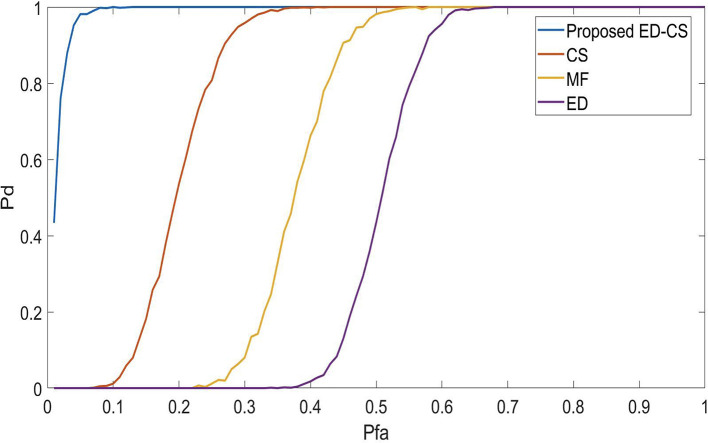
Signal-to-noise ratio (SNR) Vs probability of detection ( *P*_*d*_) in Rician channel.

In this section, we estimate the performance of the proposed ED-CS spectrum using Matlab (Matlab 2016) or Python as an alternative option ( https://jupyter.org). For the simulation, we used 20000 samples, 64-QAM, 64-FFT, overlapping factor = 4, and a NOMA waveform under a Rician channel. The simulation results offer valuable insights for analyzing concepts by providing a controlled environment to explore hypothetical scenarios. They allow researchers to observe outcomes under various conditions, offering a deeper understanding of complex systems or theories. Through simulations, one can test assumptions, validate models, and predict behaviors, aiding in decision-making processes across diverse fields such as economics, engineering, and medicine. These results enable researchers to study phenomena that are difficult or impossible to replicate in real-world settings, facilitating innovation and the refinement of theories. Ultimately, simulations serve as powerful tools for advancing knowledge and enhancing comprehension of intricate concepts. In Figure 2, we analyze the detection performance of the ED-CS and the conventional methods. Enhancing Pd in spectrum-sensing methods for smart hospitals would lead to more reliable and responsive communication networks. This improvement ensures that vital medical data and alerts are consistently detected and transmitted accurately, facilitating faster response times and critical decision-making. Patients benefit from timely care, reduced risks, and improved overall healthcare quality, making smart hospitals more effective and dependable in emergencies and routine care. Algorithms that obtain maximum detection at a low SNR are considered the best SS methods. In this case, the maximum detection is obtained at a SNR of -2 dB for the proposed ED-CS, 2.1 dB for CS, 4.2 dB for MF, and 6.2 dB for ED. Hence, it is concluded that the ED-CS outperforms the CS, MF, and ED by obtaining a gain of 4.1 dB, 6 dB, and 7.9 dB, respectively.

**Figure 3.**
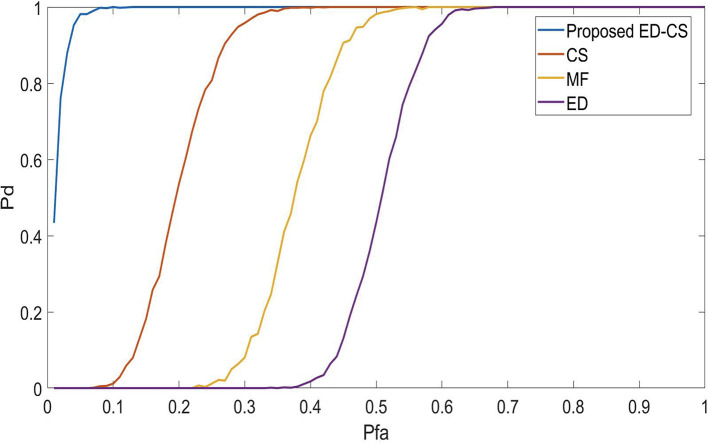
Probability of a false alarm ( *P*_*fa*_) vs probability of detection ( *P*_*d*_) under Rician channel.

The *P*_*fa*_ curves for the proposed SS technique are shown in Figure 3. If the *P*_*fa*_ is high, the algorithm misrepresents the noise as a PU signal. Reducing *P*_*fa*_ in spectrum-sensing methods within smart hospital applications would enhance the reliability of critical communication systems. This means fewer false alerts or disruptions in monitoring, ensuring seamless connectivity between medical devices and personnel. It increases patient safety, minimizes data errors, and optimizes healthcare workflows, ultimately improving the quality of care in smart hospitals. In this case, it is noted that the *P*_*d*_ is maximum at the *P*_*fa*_ of 0.1, 0.3, 0.55 and 0.62 for ED-CS, CS, MF and ED. Therefore, it is concluded that the proposed ED-CS works efficiently under high false alarm conditions compared to conventional approaches.

**Figure 4.**
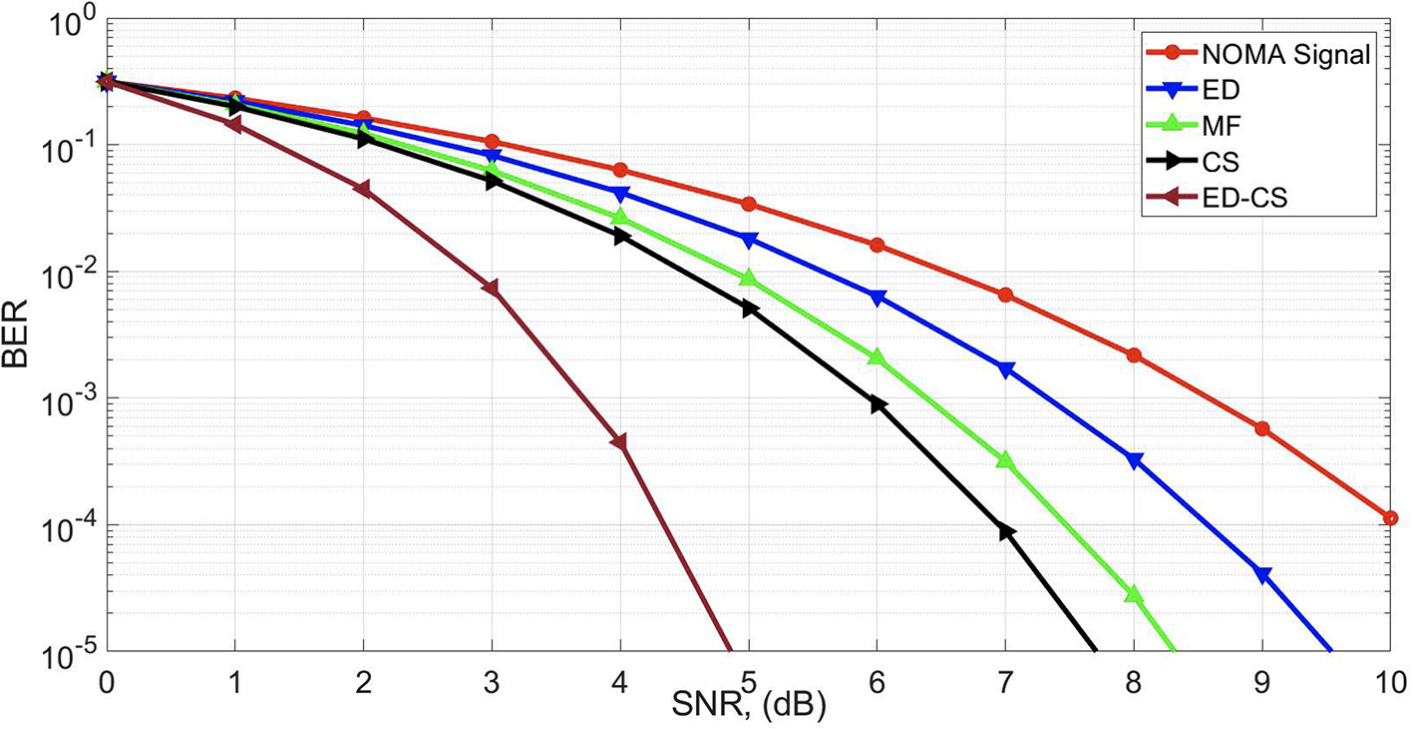
Bit error rate (BER) under Rician channel.

To analyze the throughput performance of the SS methods, a bit error rate (BER) curve was estimated, as shown in Figure 4. The main aim of this study is to optimize BER performance for a reliable framework. Enhancing the BER in smart hospital applications can significantly improve efficiency. A lower BER indicates improved data transmission reliability, which is crucial for accurate real-time communication in healthcare settings. This leads to reduced data errors in patient monitoring, diagnostics, and treatment, enabling healthcare professionals to make informed decisions promptly. Ultimately, this enhances patient care quality and overall operational efficiency in smart hospitals. The SNR gains of 2.4 dB, 3.1 dB, and 3.1 dB are obtained at the BER of 10 ^−4^ by the ED-CS as compared with the CS, MF, and ED algorithms, respectively.

**Figure 5.**
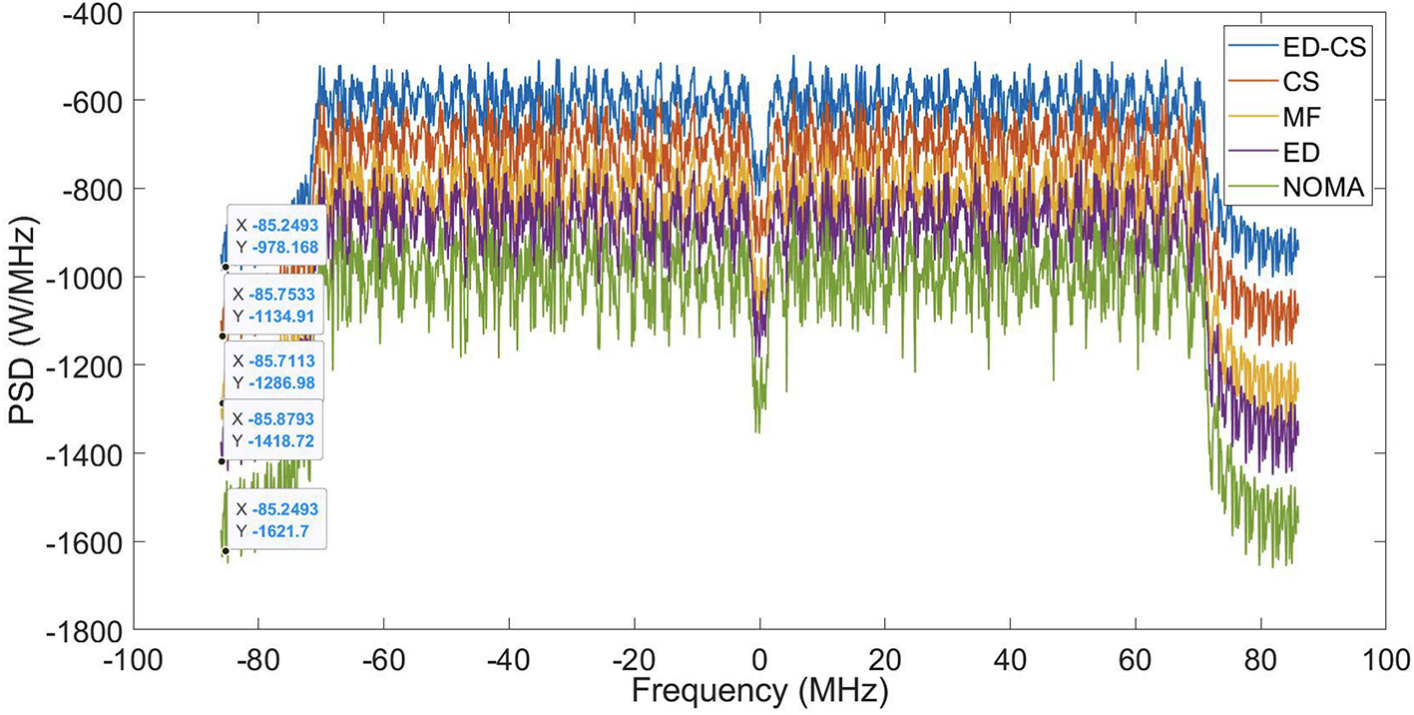
Power spectral density (PSD) performance under Rician channel.

The PSD of the NOMA waveform is shown in Figure 5. The sidelobes of NOMA are greatly reduced by the ED-CS methods, resulting in spectral efficiency. The PSD values of the NOMA waveform were -978, -1134, -1286, -1418, and -1621 for the ED, CS, MF, ED, and original NOMA waveforms, respectively. Enhancing PSD performance and utilizing SS methods in smart hospitals improve wireless communication reliability. This reduces signal interference, supports more devices, extends the battery life, and enhances patient monitoring. An enhanced PSD ensures data accuracy and enables precise diagnosis and treatment. It also ensures network reliability, enables timely responses in emergencies, improves healthcare efficiency, and ultimately enhances patient care and hospital operations.

Comparing ED, CS, MF, and ED-CS for smart healthcare applications involves considering their strengths and weaknesses in the context of specific requirements. The complex equations provided a high-level comparison of the computational loads for each method. However, the actual complexity can vary significantly depending on factors such as the specific algorithms, hardware, and software optimization used in the implementation. A brief comparison is presented in Table 2. ^22^

**Table 2.** Comparison of conventional and proposed algorithms.

Methods	Advantages	Disadvantages	Complexity
Energy detection (ED)	1. Simplicity and low complexity. 2. Suitable for wideband and unknown signal types. 3. Works in non-cooperative and dynamic environments.	1. Susceptible to high false alarm rates, especially in low SNR conditions. 2. May not be ideal for detecting weak or intermittent signals.	*O*( *N*) where *N* is the length of the received signal.
Cyclostationary spectrum (CS)	1. Effective in distinguishing between modulated and non-modulated signals. 2. Can exploit signal CS for improved detection. 3. Moderate complexity and can be adapted to various signal types.	1. Requires some knowledge of the signal's cyclic properties. 2. May still face challenges in low SNR or rapidly changing environments.	*O*( *N* ^2^)
Matched filter (MF)	1. Ideal for detecting known signals with minimal noise. 2. Provides optimal detection performance in terms of signal-to-noise ratio.	1. Limited to known signal waveforms and may not adapt well to diverse signals. 2. Complexity increases with signal diversity.	*O*( *N* ∗ *L* ∗ *K*), where *L* is the length of the filter, and *K* is the number of filters.
Energy detection-cyclostationary spectrum (ED-CS)	1. Combines the advantages of both ED and CS techniques. 2. Can adapt to a wide range of signals, making it suitable for smart healthcare applications with diverse signal sources. 3. Potentially improved detection performance compared to individual methods.	1. Increased complexity compared to individual techniques. 2. Requires careful design and parameter tuning.	*O*( *N* ^3^)

## 5. Conclusions

In this article, we propose a hybrid ED-CS algorithm to enhance and optimize the spectral performance of the 6G NOMA waveform. Under a Rician channel, the proposed ED-CS method is compared to the traditional CS, ED, and MF methods. It can be seen that the ED-CS method does better than the traditional SS methods in terms of Pd, Pfa, BER, and PSD. It is seen that the ED-CS obtained efficient signal detection at low SNR and was also able to minimize the Pfa effect. To address the critique regarding implementation challenges and computational resources required by the new method, it’s crucial to provide a comprehensive analysis, which we have done in the paper. In the proposed article, we are analyzing the performance of the proposed algorithm for the smart health care system. We have analyzed the probability of detection, false alarm, BER, PSD, and complexity. Further, we have compared the other algorithms with the proposed one, and it is evident that the proposed algorithm shows a substantial improvement as compared with the conventional methods. The proposed solutions for 6G-based smart hospital applications offer numerous advantages. The utilization of these methodologies facilitates highly dependable and minimally delayed communication, which is of utmost importance in the context of remote surgical procedures and the continuous monitoring of patients in real-time. The proposed hybrid spectrum sensing methods in 6G-based smart healthcare applications face limitations, including increased computational complexity and energy consumption due to the integration of multiple sensing techniques. Moreover, hybrid sensing may require sophisticated hardware and signal processing algorithms, potentially increasing system cost and complexity. To overcome these challenges, future studies can focus on developing more efficient algorithms and hardware implementations tailored to the specific requirements of healthcare applications. Additionally, advancements in machine learning and artificial intelligence can be leveraged to optimize hybrid sensing performance while minimizing computational overhead and energy consumption, ensuring cost-effective and scalable solutions for smart healthcare systems.

## Data Availability

No data are associated with this article.
