# Supplementary material for: Exploring and analyzing the role of hybrid spectrum sensing methods in 6G-based smart health care applications
Source: F1000Res. 2024 May 23;13:110. Originally published 2024 Feb 19. [Version 2] doi: 10.12688/f1000research.144624.2 (PMC11184273; doi:10.12688/f1000research.144624.2)
Supplement: Peer review history

# Reviewer report: Reviewer response for version 2

**DOI:** 10.5256/f1000research.166382.r281950

**Published:** 2024-06-17

**Authors:** H. ALSHARIF

**Affiliations:** 1 Korea University (Ringgold ID: 34973), Seongbuk-gu, Seoul, South Korea

The comments for my enquires are available online for version article 1. However, after reviewing the revised version of the article, I see that the authors have addressed the comments. The article has improved and now meets the academic requirements for indexing.

Is the rationale for developing the new method (or application) clearly explained?

Yes

Is the description of the method technically sound?

Partly

Are the conclusions about the method and its performance adequately supported by the findings presented in the article?

Yes

If any results are presented, are all the source data underlying the results available to ensure full reproducibility?

Yes

Are sufficient details provided to allow replication of the method development and its use by others?

Partly

Reviewer Expertise:

Wireless communications

I confirm that I have read this submission and believe that I have an appropriate level of expertise to confirm that it is of an acceptable scientific standard.

# Reviewer report: Reviewer response for version 2

**DOI:** 10.5256/f1000research.166382.r281949

**Published:** 2024-06-06

**Authors:** Fahad Ahmad

**Affiliations:** 1 School of Computing, University of Portsmouth, Portsmouth, England, UK

Authors have made suggested changes sufficiently and clarified the doubts.

Is the rationale for developing the new method (or application) clearly explained?

Partly

Is the description of the method technically sound?

Partly

Are the conclusions about the method and its performance adequately supported by the findings presented in the article?

No

If any results are presented, are all the source data underlying the results available to ensure full reproducibility?

No source data required

Are sufficient details provided to allow replication of the method development and its use by others?

Partly

Reviewer Expertise:

smart healthcare applications, artificial intelligence, quantum computing

I confirm that I have read this submission and believe that I have an appropriate level of expertise to confirm that it is of an acceptable scientific standard.

# Reviewer report: Reviewer response for version 1

**DOI:** 10.5256/f1000research.158445.r263432

**Published:** 2024-05-07

**Authors:** H. ALSHARIF

**Affiliations:** 1 Korea University (Ringgold ID: 34973), Seongbuk-gu, Seoul, South Korea

Researchers are focused on quick and real-time healthcare and monitoring systems because of the contemporary modern world’s rapid technological improvements. Utilizing the primary user (PU) spectrum, cognitive radio (CR) can be highly useful for efficient and intelligent healthcare systems to send and receive patient health data. This paper deals with a hot area of investigation at the moment. I have looked at the mathematics and it looks sound. I would have liked to have seen a longer conclusion that discussed the effects of the various methods investigated. At this point, I recommend these parts be revised again and be expressed more analytically in a revised version.  

 1. Section 1.2 (1.2. Contributions) The contribution points should be summarized in 4 points instead of 7 points be sharp and narrow and reflect the content and the results well.

 2. Table 2 (validation table) The comparison of the proposed technique is given based on which studies in the literature.

 3. The authors did not describe well how spectrum sensing plays a crucial role in enabling smart healthcare applications in the context of 6G networks.

 3. What are the primary challenges faced by healthcare applications in terms of spectrum availability and efficiency, and how can hybrid spectrum sensing address these challenges?

 4. The potential benefits of integrating hybrid spectrum sensing methods into 6G-based smart healthcare systems were not explained well; and deserve more discussion.

 5. Section 5. (Conclusion) should discuss the limitations and disadvantages of the proposed technique and how this can be overcome by this shortcoming in future studies.

Is the rationale for developing the new method (or application) clearly explained?

Yes

Is the description of the method technically sound?

Partly

Are the conclusions about the method and its performance adequately supported by the findings presented in the article?

Yes

If any results are presented, are all the source data underlying the results available to ensure full reproducibility?

Yes

Are sufficient details provided to allow replication of the method development and its use by others?

Partly

Reviewer Expertise:

Wireless communications

I confirm that I have read this submission and believe that I have an appropriate level of expertise to confirm that it is of an acceptable scientific standard, however I have significant reservations, as outlined above.

# Response

**Published:** 2024-05-24

**Authors:** Arun Kumar

**Affiliations:** New Horizon College of Engineering, India

Author Response for Reviewer 2 Peer Review Report:

**Reviewer Comments:** Researchers are focused on quick and real-time healthcare and monitoring systems because of the contemporary modern world’s rapid technological improvements. Utilizing the primary user (PU) spectrum, cognitive radio (CR) can be highly useful for efficient and intelligent healthcare systems to send and receive patient health data. This paper deals with a hot area of investigation at the moment. I have looked at the mathematics and it looks sound. I would have liked to have seen a longer conclusion that discussed the effects of the various methods investigated. At this point, I recommend these parts be revised again and be expressed more analytically in a revised version.  

 1. Section 1.2 (1.2. Contributions) The contribution points should be summarized in 4 points instead of 7 points be sharp and narrow and reflect the content and the results well.

**Author Ans. Thank You Sir. It is updated as given below:**

1. The hybrid approach provides superior SS by leveraging the sensitivity of ED to detect energy in a frequency band and the precision CS to confirm the presence of specific signals. This results in a comprehensive and accurate view of the spectrum environment within the smart hospital. High probability of detection in 6G-based smart healthcare applications is crucial for seamless, ultra-reliable connectivity. These algorithms minimize interference, enabling real-time, high-quality data transmission from medical devices. This enhances telemedicine, remote patient monitoring, and precision diagnostics, ultimately improving healthcare accessibility and patient outcomes in the advanced 6G era.
2. The hybrid ED-CS ensures the reliable detection of wireless medical devices and sensors. This is crucial for monitoring patients' vital signs, enabling timely interventions, and enhancing patient safety. With accurate spectrum sensing, smart hospitals can optimize their wireless networks and devices, leading to improved operational efficiency, reduced downtime, and cost savings.
3. As 6G technology evolves, the hybrid approach ensures that smart hospitals are well-equipped to adapt to the increasing demands of advanced wireless communication. It future-proofs healthcare infrastructure, allowing hospitals to stay at the forefront of technology by enhancing the throughput of the framework. High-throughput SS algorithms in 6G-based smart healthcare applications facilitate rapid data transmission, supporting real-time remote monitoring and medical data exchange. This enables complex diagnostic procedures, telemedicine, and seamless healthcare services, offering enhanced patient care and health outcomes in the high-speed, low-latency 6G environment.
4. Real-time and accurate spectrum sensing enhances patient care by enabling continuous monitoring and data transmission from medical devices. This contributes to early detection of medical issues, faster response times, and better overall patient outcomes.

2. **Reviewer Comments:** Table 2 (validation table) The comparison of the proposed technique is given based on which studies in the literature.

**Author Ans. **Thank You Sir: it is given as below:

 Table 2 presents a validation of the proposed technique by comparing it with previous studies in the literature. This comparison serves as a benchmark to evaluate the effectiveness and performance of the proposed approach in addressing similar problems:

 3. **Reviewer Comments: **The authors did not describe well how spectrum sensing plays a crucial role in enabling smart healthcare applications in the context of 6G networks.

**Author Ans.** Thank You Sir. It is revised in the proposed work:

 In the framework of 6G networks, spectrum sensing is essential for developing smart healthcare applications. 6G-enabled healthcare systems may dynamically distribute spectrum resources to medical equipment, guaranteeing uninterrupted connectivity and real-time data transfer, by effectively identifying and analyzing underutilized or unused spectrum bands. This feature makes it easier for telemedicine, IoT devices for healthcare, and remote patient monitoring to proliferate, promoting prompt and accurate healthcare delivery. Additionally, spectrum sensing improves spectrum reliability and efficiency, which is essential for enabling the ultra-low latency and large data rates needed by developing healthcare applications in 6G networks [6].

 Further motivations are also given which address the above question:

***1.1. Motivation***

 SS plays a crucial role in enhancing healthcare applications across various scenarios by ensuring reliable and efficient wireless communication. Here are different scenarios where spectrum sensing benefits healthcare [7-9]:

- Emergency Response and Disaster Recovery: During natural disasters or emergencies, dedicated spectrum sensing helps prioritize and allocate spectrum resources for healthcare providers. This ensures that first responders and medical teams have uninterrupted communication for coordinating rescue efforts and providing critical medical assistance, even in congested or disrupted networks.
- Wireless Medical Devices: Spectrum sensing is essential for wireless medical devices like remote patient monitors, wearable health trackers, and smart healthcare implants. These devices rely on spectrum sensing to identify the most suitable and interference-free frequency bands, ensuring real-time data transmission and accurate patient monitoring.
- Telemedicine: Spectrum sensing is vital for telemedicine applications, where doctors and patients communicate remotely. It enables healthcare professionals to maintain consistent and high-quality video and audio connections, even in crowded wireless environments. This ensures that telemedicine consultations and remote diagnostics are conducted without interruptions, improving the accessibility of healthcare services.
- IoT Healthcare Devices: The IoT has revolutionized healthcare with devices such as smart pill dispensers, medication trackers, and patient location monitors. Spectrum sensing ensures these IoT devices can operate in less congested bands, facilitating the reliable transmission of health-related data to healthcare providers and caregivers.
- Wireless Imaging and Diagnostics: Healthcare facilities increasingly rely on wireless technologies for transmitting large medical imaging files, including MRIs, CT scans, and X-rays. Spectrum sensing helps allocate suitable spectrum resources, ensuring that these critical files are transmitted efficiently and promptly, allowing for quicker diagnosis and treatment planning.
- Mobile Clinics: Spectrum sensing is beneficial for mobile healthcare clinics that operate in remote or underserved areas. These clinics can use spectrum sensing to identify the best available spectrum for their wireless communication needs, ensuring that healthcare professionals can access patient records, medical databases, and telemedicine consultations without network disruptions.
- Healthcare Robotics: Spectrum sensing is essential for healthcare robotics, including robotic surgical systems and remote-controlled medical robots. It ensures precise and real-time control during medical procedures, where any signal interference or delay can have critical consequences.
- Ambulance Communication: Ambulances equipped with advanced medical equipment and communication systems rely on spectrum sensing. It helps these vehicles maintain connectivity, allowing paramedics and emergency medical personnel to transmit patient information, vital signs, and other critical data to hospitals, ensuring seamless patient care transitions.

 

 3. **Reviewer Comments: ** What are the primary challenges faced by healthcare applications in terms of spectrum availability and efficiency, and how can hybrid spectrum sensing address these challenges?

**Author Ans. **Thank You Sir. It is included in the introduction section as:

 Healthcare applications encounter significant challenges regarding spectrum availability and efficiency. The increasing demand for wireless healthcare services exacerbates spectrum congestion, leading to potential interference and degraded performance. Moreover, strict regulatory constraints limit the available spectrum for medical use. Hybrid spectrum sensing presents a promising solution by combining multiple sensing techniques, such as energy detection, matched filtering, and cyclostationary feature detection. By leveraging the strengths of each method, hybrid sensing enhances spectrum awareness, enabling healthcare systems to detect and utilize idle spectrum more effectively. This approach also improves spectrum utilization efficiency by accurately identifying and dynamically accessing available spectrum resources while mitigating interference risks. Additionally, hybrid spectrum sensing enhances reliability by providing robust detection in diverse environmental conditions and mitigating the effects of fading and shadowing. Overall, hybrid spectrum sensing offers a comprehensive solution to address the spectrum-related challenges faced by healthcare applications, ensuring reliable and efficient wireless connectivity for critical medical services [3].

 4. **Reviewer Comments:** The potential benefits of integrating hybrid spectrum sensing methods into 6G-based smart healthcare systems were not explained well; and deserve more discussion.

**Author Ans.** Thank You Sir. It is included in the beginning of literature section as given below:

 Integrating hybrid spectrum sensing methods into 6G-based smart healthcare systems offers a multitude of benefits. Firstly, it enhances spectrum efficiency by intelligently utilizing available spectrum resources, ensuring optimal allocation for healthcare applications amidst growing demand and spectrum scarcity. Secondly, hybrid sensing improves reliability by mitigating interference and dynamically adapting to changing wireless environments, thereby enhancing the quality and consistency of healthcare data transmission. Thirdly, it enables seamless connectivity and coverage extension, crucial for supporting remote patient monitoring, telemedicine, and IoT devices in diverse healthcare settings. Additionally, hybrid spectrum sensing enhances security and privacy by identifying and mitigating potential threats and unauthorized access attempts. Moreover, by optimizing spectrum usage and improving connectivity, it facilitates innovative healthcare services and applications, fostering advancements in medical diagnostics, treatments, and patient care delivery. Overall, the integration of hybrid spectrum sensing into 6G-based smart healthcare systems promises to revolutionize healthcare delivery, ensuring efficient, reliable, and secure wireless communication for improved patient outcomes.

 5. **Reviewer Comments:** Section 5. (Conclusion) should discuss the limitations and disadvantages of the proposed technique and how this can be overcome by this shortcoming in future studies.

**Author Ans.** Thank You Sir, it is included in the conclusion section as:

 The proposed hybrid spectrum sensing methods in 6G-based smart healthcare applications face limitations, including increased computational complexity and energy consumption due to the integration of multiple sensing techniques. Moreover, hybrid sensing may require sophisticated hardware and signal processing algorithms, potentially increasing system cost and complexity. To overcome these challenges, future studies can focus on developing more efficient algorithms and hardware implementations tailored to the specific requirements of healthcare applications. Additionally, advancements in machine learning and artificial intelligence can be leveraged to optimize hybrid sensing performance while minimizing computational overhead and energy consumption, ensuring cost-effective and scalable solutions for smart healthcare systems.

# Reviewer report: Reviewer response for version 1

**DOI:** 10.5256/f1000research.158445.r263428

**Published:** 2024-04-17

**Authors:** Fahad Ahmad

**Affiliations:** 1 School of Computing, University of Portsmouth, Portsmouth, England, UK

Minor:

- Introduces a novel ED and CS spectrum sensing combination for cognitive radio in smart healthcare, enhancing precision in data transmission.
- Relevant to 6G technology applications in healthcare, addressing the need for quick, real-time monitoring systems.

Major:

- The study primarily utilizes simulations for testing, which raises concerns about its relevance to real-world scenarios. Simulations, although useful, might not fully capture the complexities and unpredictabilities encountered in practical environments, leading to potential gaps in the method's applicability and effectiveness when applied outside a controlled setting. 
- There is a lack of in-depth comparative analysis with established traditional methods like Membrane Filtration (MF), Electrodialysis (ED), and Crystallization (CS). Such analysis is crucial for understanding how the new method stands against these conventional techniques in terms of efficiency, cost-effectiveness, and potential limitations, particularly in practical applications.
- The discussion around the implementation challenges and the computational resources required by the new method is insufficient. This omission raises questions about its feasibility in real-world applications. Understanding the computational demands and logistical hurdles is essential for assessing the method's viability and scalability.
- While the conclusion acknowledges the potential for future improvements, it falls short of a comprehensive discussion on the method's current limitations and areas needing further research. This oversight restricts the study's perspective on how the method can be refined and applied more broadly, limiting its contribution to the field's advancement.

Is the rationale for developing the new method (or application) clearly explained?

Partly

Is the description of the method technically sound?

Partly

Are the conclusions about the method and its performance adequately supported by the findings presented in the article?

No

If any results are presented, are all the source data underlying the results available to ensure full reproducibility?

No source data required

Are sufficient details provided to allow replication of the method development and its use by others?

Partly

Reviewer Expertise:

6G, smart healthcare applications, artificial intelligence, quantum computing

I confirm that I have read this submission and believe that I have an appropriate level of expertise to confirm that it is of an acceptable scientific standard, however I have significant reservations, as outlined above.

# Response

**Published:** 2024-04-17

**Authors:** Arun Kumar

**Affiliations:** New Horizon College of Engineering, India

Thank you for giving me the opportunity to answer the inquiries. Please find below the response to the comment given:

 1. **The study primarily utilizes simulations for testing, which raises concerns about its relevance to real-world scenarios. Simulations, although useful, might not fully capture the complexities and unpredictabilities encountered in practical environments, leading to potential gaps in the method's applicability and effectiveness when applied outside a controlled setting.**

**Ans. Thank You Sir. **Simulation results offer valuable insights for analyzing concepts by providing a controlled environment to explore hypothetical scenarios. They allow researchers to observe outcomes under various conditions, offering a deeper understanding of complex systems or theories. Through simulations, one can test assumptions, validate models, and predict behaviors, aiding in decision-making processes across diverse fields such as economics, engineering, and medicine. These results enable researchers to study phenomena that are difficult or impossible to replicate in real-world settings, facilitating innovation and the refinement of theories. Ultimately, simulations serve as powerful tools for advancing knowledge and enhancing comprehension of intricate concepts.

 2. **There is a lack of in-depth comparative analysis with established traditional methods like Membrane Filtration (MF), Electrodialysis (ED), and Crystallization (CS). Such analysis is crucial for understanding how the new method stands against these conventional techniques in terms of efficiency, cost-effectiveness, and potential limitations, particularly in practical applications.**

**Ans. Thank you, sir**. We have not used the above terms (membrane filtration (MF), electrodialysis (ED), and crystallization (CS)) in our paper. In our paper, ED = Energy Detection, MF=Matched Filter, and CS = Cyclostationary Spectrum Sensing, which are all well defined in the paper, please see Table 2.

 3. **The discussion around the implementation challenges and the computational resources required by the new method is insufficient. This omission raises questions about its feasibility in real-world applications. Understanding the computational demands and logistical hurdles is essential for assessing the method's viability and scalability.**

**Ans. Thank You, sir.** To address the critique regarding implementation challenges and computational resources required by the new method, it's crucial to provide a comprehensive analysis, which we have done in the paper. In the proposed article, we are analyzing the performance of the proposed algorithm for the smart health care system. We have analyzed the probability of detection, false alarm, BER, PSD, and complexity. Further, we have compared the other algorithms with the proposed one, and it is evident that the proposed algorithm shows a substantial improvement as compared with the conventional.

 4. **While the conclusion acknowledges the potential for future improvements, it falls short of a comprehensive discussion on the method's current limitations and areas needing further research. This oversight restricts the study's perspective on how the method can be refined and applied more broadly, limiting its contribution to the field's advancement.**

**Ans. Thank you**, sir. In Table . 2, we have already cited the limitations of the proposed algorithms based on simulation results. i.e., 1. increased complexity compared to individual techniques; and 2. requires careful design and parameter tuning. Further, the following can be added: A limitation of the proposed hybrid spectrum sensing method is the increase in complexity, which compromises the reliability of real-time healthcare data transmission and communication due to the dynamic design and parameters of the framework. Further we are "Exploring and analyzing the role of hybrid spectrum sensing methods in 6G-based smart health care applications". We are not claiming to design of hardware model so we have no simulations.
